# Organelle Genetics in Plants

**DOI:** 10.3390/ijms22042104

**Published:** 2021-02-20

**Authors:** Pedro Robles, Víctor Quesada

**Affiliations:** Instituto de Bioingeniería, Campus de Elche, Universidad Miguel Hernández, 03202 Elche, Spain; probles@umh.es

## Abstract

Eleven published articles (4 reviews, 7 research papers) are collected in the Special Issue entitled “Organelle Genetics in Plants.” This selection of papers covers a wide range of topics related to chloroplasts and plant mitochondria research: (i) organellar gene expression (OGE) and, more specifically, chloroplast RNA editing in soybean, mitochondria RNA editing, and intron splicing in soybean during nodulation, as well as the study of the roles of transcriptional and posttranscriptional regulation of OGE in plant adaptation to environmental stress; (ii) analysis of the nuclear integrants of mitochondrial DNA (NUMTs) or plastid DNA (NUPTs); (iii) sequencing and characterization of mitochondrial and chloroplast genomes; (iv) recent advances in plastid genome engineering. Here we summarize the main findings of these works, which represent the latest research on the genetics, genomics, and biotechnology of chloroplasts and mitochondria.

In plant cells, most DNA is located in the nucleus, although chloroplasts and mitochondria also contain part of the genetic material. The organization and inheritance patterns of this organellar DNA are quite different to that of nuclear DNA. The presence of DNA in chloroplasts and mitochondria reveals their evolutionary origin. Considerable phylogenetic evidence supports the hypothesis that both organelles come from ancestral free-living prokaryotes, which established an endosymbiotic relationship with a primitive eukaryotic cell [1,2,3]. The vast majority of the genes of ancestral prokaryotes were transferred to the nucleus of the host cell during the course of evolution. Consequently, present-day chloroplast and mitochondrial genomes contain only between 100 and 200 genes that encode proteins required for ATP synthesis, photosynthesis, and gene expression, including RNA synthesis, processing, and translation. Nevertheless, chloroplasts and mitochondria harbor several thousands of proteins, and most are encoded by the nucleus, translated in the cytoplasm, and transported to their target organelle. As a result, the expression of nuclear and organellar genomes has to be very precisely coordinated, which is achieved mainly in the organelles at the posttranscriptional level [4].

In this Special Issue “Organelle Genetics in Plants,” 11 articles were accepted with four reviews and seven original research articles covering outstanding advances in different chloroplast and plant mitochondria research fields (Table 1). These works focus on topics related to organellar gene expression (OGE) (chloroplast RNA editing in soybean [5], mitochondria RNA editing and intron splicing in soybean during nodulation [6] and the roles of transcriptional and post-transcriptional regulation of OGE in responses to environmental stress [7,8]); the analysis of nuclear integrants of mitochondrial DNA (NUMTs) or plastid DNA (NUPTs) [9]; the sequencing and characterization of organellar genomes (the mitogenomes of common bean [10] and four Trifolium species [11], and the chloroplast genomes (plastomes) of *Trentepohlia odorata* [12], three *Utricularia amethystina* morphotypes [13], and three plant parasitic *Macrosolen* species [14]); and finally, the most recent advances in plastid genome engineering [15]. In this editorial, we sum up the main findings of these eleven insightful manuscripts.

RNA editing is a posttranscriptional process that changes the RNA sequence of land plant chloroplasts and mitochondrial transcripts insofar as the information in genomic DNA differs from that in mature RNA. Two research papers in this Special Issue analyze RNA editing in soybean chloroplasts [5] and mitochondria [6]. In the first article, Zhu et al. [5] report the characterization of the soybean *yellow leaf* (*yl*) mutant which displays chlorophyll defects and abnormal photosynthesis. Fine mapping of the *YL* gene reveals that it encodes the chloroplast-located Organelle RNA Recognition Motif-Containing Protein 1 (GmORRM1), orthologous of the characterized Arabidopsis AtORRM1 and maize ZmORRM1 proteins, which strongly suggests that YL functions in RNA editing. To advance the function of YL, Zhu et al. [5] report performing DNA resequencing and RNA sequencing (RNA-seq); they identified in the wild type (WT) 44-predicted soybean chloroplast editing sites, most of which lead to alterations to the encoded amino acids. By comparing RNA editing between WT and *yl* leaves, Zhu et al. [5] found alterations in the editing levels of 19 sites distributed in 12 chloroplast transcripts, and encoding components of the Clp protease proteolytic subunit, NADH dehydrogenase-like (NDH) complex, cytochrome *b_6_f* complex, photosystem II (PSII) complex, RNA polymerase, and ribosomal proteins. These findings suggest that the effect of the *yl* mutation on editing is site-specific, but not transcript-specific. Zhu et al. [5] propose that reducing the RNA editing of *petB-611* and *psbL-2*, respectively encoding one of the major subunits of the cytochrome *b_6_f* complex (mediating electron transfer between PSII and I) and a conserved low-molecular-weight protein of PSII, would be the major contributors to the *yl* phenotype. Therefore, soybean YL protein influences photosynthesis, possibly by its function in chloroplast RNA editing. 

Following this crop, soybean has the ability to perform symbiotic nitrogen fixation in root nodules. In the second work, Sun et al. [6] examine how the levels of RNA editing in soybean mitochondrial transcripts are affected by nodule formation to investigate its possible biological purpose. To this end, RNA-seq was performed using total RNA extracted from collected nodules (N) after rhizobium inoculation, stripped roots (SR), and uninoculated roots (UR). These authors identified 631 RNA editing sites with at least 15% edited transcripts in all three biological replicates of any one of the tested tissues (N, SR and UR), but only 12% of these sites were differentially edited between any two of the three samples. One of the mitochondrial transcripts that underwent extensive RNA editing was the *matR* transcript, which encodes an intron maturase that mediates group-II intron splicing. The splicing efficiencies of 20 mitochondrial introns were analyzed by qRT-PCR among the N, SR, and UR samples. Sun et al. [6] found that *nad1* introns 2/3/4, *nad4* intron 3, *nad5* introns 2/3, *cox2* intron 1, and *ccmFc* intron 1 splicing efficiencies were higher in the N and SR than in the UR samples. Moreover, the splicing efficiency of *nad4* intron 1 was more greatly enhanced in N than in UR and SR. Consistently, higher protein levels of NAD4 were observed in the N samples than in the other two, which affected I+III2 mitochondrial supercomplex formation during nodulation. The greater abundance of *matR* transcripts in the N and SR samples than in the UR samples prompted Sun et al. [6] to propose that the enhanced splicing efficiencies of the aforementioned introns could be due to an increase in the levels of *matR* transcripts and/or to changes in RNA editing. Nevertheless, a causal relationship between these observations requires further investigation.

Two reviews are about the emerging importance of OGE processes in plant responses to environmental stress. Quesada and Robles [7] and Zhang et al. [8] compile the results hitherto published from analyzing plant mutants, mainly in Arabidopsis and rice, revealing a connection between OGE and tolerance to environmental stress. Our review [7] summarizes the works reporting the phenotypic and molecular analyses of plant mutants exhibiting altered sensitivity to salinity that are affected in nuclear genes involved in OGE regulation at transcriptional or post-transcriptional levels. We conclude that the detailed characterization of these mutants strongly supports a link between OGE and plant salt tolerance, likely through organelle-to-nucleus signaling, and highlights the important role of chloroplast and mitochondrion homeostasis in plant adaptation to salinity. Following a similar approach, Zhang et al. [8] focus on the published works reporting a link between plant environmental stress responses and: (i) the transcriptional control of chloroplast gene expression; (ii) RNA metabolism in chloroplast; (iii) translation in this organelle. Together these studies indicate that chloroplast gene expression is important for plant stress responses, and the authors propose that novel tools like CRISPR/Cas9, RNA interference, and artificial RNA editing systems for carrying out the RNA editing of specific sites in chloroplasts should be developed to better investigate the molecular mechanisms of chloroplast gene expression in response to environmental cues. 

The transfer of genetic material from plastids and mitochondria to the nucleus respectively gives rise to NUPTs and NUMTs. The comprehensive review paper by Zhang et al. [9] analyzes and summarizes the recent advances made in the characterization and distribution patterns of organellar DNA-derived sequences in the plant nuclear genomes, the genetic consequences and fate of plant NUPTs/NUMTs, their effects on the nuclear genome structure and evolution, as well as mechanisms of organellar DNA integration. Zhang et al. [9] report the important role that integrated organellar DNAs play in increasing genetic diversity, promoting gene and genome evolution, and sex chromosome evolution in dioecious plants. Notwithstanding, these authors also highlight the still unanswered questions about NUMTs/NUPTs, such as the precise mechanism of organellar DNA transfer, the effects of organellar DNA transfer on gene activity regulation, and the caused genome instability and the defense mechanisms elicited by organellar DNA transfer to the nucleus.

Two research manuscripts report the sequencing and assembly of the complete mitogenomes of the common bean (*Phaseolus vulgaris*) [10] and four *Trifolium* species from subgenera *Chronosemium* (*T. aureum* and *T. grandiflorum*) and *Trifolium* (*T. meduseum* and *T. pratense*) [11]. All these species belong to Fabaceae, an economically and ecologically important family of flowering plants. In these works, the authors analyzed the gene content, size, and repeat structure of assembled mitogenomes and performed different phylogenetic analyses. The sequencing of the *P. vulgaris* mitogenome by Bi et al. [10] reveals differential selective pressure on protein-coding genes (PCGs) and 486 predicted RNA-editing sites in the PCGs, all of which are C-to-U conversions, which may generate initiation, termination, or internal codons with totally unpredictable functions. This work paves the way to conduct further genomic breeding studies in common bean, and provides valuable information for future evolutionary and molecular studies of leguminous plants. The sequenced mitogenomes of *Trifolium* allowed Choi et al. [11] to perform comparative analyses of genome evolution for three plant cellular compartments (mitochondrion, nucleus, and plastid) to improve our understanding of how these genomes evolved in *Trifolium*. Choi et al. [11] also identified in *Trifolium* an independent and novel gene fission event of the *ccmFn* gene caused by a 59 bp deletion, as well as intracellular gene transfer events in Fabaceae. 

Three works in the Special Issue take advantage of chloroplast genome sequencing and ulterior analyses, such as the determination of plastome structure, gene content and genetic diversity, and RNA editing and phylogenetic studies. This allows an in-depth identification, classification, and evolution of species with limited reports to date. 

Zhu et al. [12] report for the first time, the whole chloroplast genome of a species of the Trentepholiales order of Chlorophyta algae, *Trentepohlia odorata*. *T. odorata* plastome is circular, 399,372 bp long, with the typical quadripartite structure. It harbors 63 protein-coding genes as well as 31 tRNA and 3 rRNA genes. This is the largest plastome currently identified in the Ulvophyceae class of green algae. These authors also performed phylogenetic analyses based on the chloroplast genome to shed light on the evolution of Ulvophyceae. They report that Trentepohliales are nested with Bryopsidales and Dasycladales, and are closely related to the latter. 

Silva et al. [13] sequenced the complete genome of three morphotypes with different colored flowers (yellow, white, and purple) of *Utricularia amethystina*, a terrestrial species belonging to the Lentibulariaceae family of carnivorous plants. The three plastomes are similar in size, about 150,000 bp, show the archetypal quadripartite structure of angiosperms, and have a similar number of annotated genes (137). In addition, the different number of repeats and chloroplast microsatellites among the three morphotypes, and the exclusive inversion of the *petN* and *psbM* genes position in the yellow one, reveal intraspecific genetic variability. The phylogenetic analysis of 15 chloroplast genomes of Lentibulariaceae specimens showed that the *Utricularia* genus is monophyletic, being the yellow morphotype, sister to the purple, and sharing a common ancestor with the white one. This work also sheds light on the evolution of photosynthesis in the Lentibulariaceae family. Although it had been previously proposed that terrestrial species of *Utricularia* might have lost the *ndh* complex genes, which occur in *U. reniformis*, the fact that the three *U. amesthystina* morphotypes retain all the *ndh* genes refutes this hypothesis, and suggests that *ndhs* in terrestrial Utricularia were independently lost and regained.

In the last work on plastome characterization, Nie et al. [14] focus on three species of the plant parasitic genus *Macrosolen* (*M. cochinchinensis*, *M. tricolor,* and *M*. *bibracteolatus*) that have extremely similar morphologies, but different medicinal effects. The three plastomes exhibit the classic quadripartite structure, are similar in size ranging from 126,621 (*M. tricolor*) to 129,570 bp (*M. cohinchinensis*), and include 111 genes (68 protein coding, 35 tRNAs, and 8 rRNAs) and 2 pseudogenes (*ycf1* and *rpl2*). The phylogenetic analyses using 58 common protein-coding genes of 16 species or the *matK* genes from 15 species of the Santalales order gave similar results: the three *Macrosolen* species are gathered in one branch, whereas the Loranthaceae, to which the *Macrosolen* genus belongs, and Viscaceae families are monophyletic clades.

Finally, Yu et al. [15] review the current status of plastid transformation, a technical approach that is drawing more attention in order to develop new genetically engineered crops. Among the benefits of chloroplast transformation vs. nuclear transformation, the many copies of the plastome that chloroplasts contain, and the many chloroplasts that a plant cell usually harbors, must be highlighted. Hence higher protein accumulation levels can often be achieved when transgenes are inserted into the plastome and not into the nuclear genome. However, several technical difficulties make the process more challenging. This article reports the current methods and recent advances in the three main steps to achieve plastid transformation: (i) DNA delivery to the chloroplasts of plant cells by biolistic transformation, PEG-mediated transfection or, more recently, by nanoparticles; (ii) DNA insertion into the plastome through homologous recombination, which can be stimulated by using recent genome editing tools, such as CRISPR-Cas; (iii) regeneration of transplastomic cells and plants. The authors finally reviewed the agronomic traits hitherto engineered in crops by this approach and its potential applications. 

Overall, the contributions published in this Special Issue (Table 1) illustrate recent advances and diverse insights into the field of organelle genetics in plants. We wish to thank all the authors for their contributions and the reviewers for their critical assessments of these articles. We also thank the assistant editor Ms. Chaya Zeng for giving us the opportunity to serve as guest editors of the Special Issue “Organelle Genetics in Plants.”

## Figures and Tables

**Table 1:** Contributors to the Special Issue “Organelle Genetics in Plants”.

Authors	Title	Type
Zhu et al. [5]	Mutation of *YL* Results in a Yellow Leaf with a Chloroplast RNA Editing Defect in Soybean	Research
Sun et al. [6]	Differential RNA Editing and Intron Splicing in Soybean Mitochondria during Nodulation	Research
Robles and Quesada [7]	Transcriptional and Post-transcriptional Regulation of Organellar Gene Expression (OGE) and Its Roles in Plant Salt Tolerance	Review
Zhang et al. [8]	The Role of Chloroplast Gene Expression in Plant Responses to Environmental Stress	Review
Zhang et al. [9]	Nuclear Integrants of Organellar DNA Contribute to Genome Structure and Evolution in Plants	Review
Bi et al. [10]	Characterization and Analysis of the Mitochondrial Genome of Common Bean (*Phaseolus vulgaris*) by Comparative Genomic Approaches	Research
Choi et al. [11]	Comparative Mitogenome Analysis of the Genus *Trifolium* Reveals Independent Gene Fission of *ccmFn* and Intracellular Gene Transfers in Fabaceae	Research
Zhu et al. [12]	Characterization of the Chloroplast Genome of *Trentepohlia odorata* (Trentepohliales, Chlorophyta) and Discussion of its Taxonomy	Research
Silva et al. [13]	Intraspecific Variation within the *Utricularia amethystina* Species Morphotypes Based on Chloroplast Genomes	Research
Nie et al. [14]	Gene Losses and Variations in Chloroplast Genome of Parasitic Plant *Macrosolen* and Phylogenetic Relationships within Santalales	Research
Yu et al. [15]	Plastid Transformation: How Does it Work? Can it Be Applied to Crops? What Can it Offer?	Review
